# Synthesis of Norbornene Derived Helical Copolymer by Simple Molecular Marriage Approach to Produce Smart Nanocarrier

**DOI:** 10.1038/srep44857

**Published:** 2017-03-22

**Authors:** Shivshankar R. Mane, Ashlin Sathyan, Raja Shunmugam

**Affiliations:** 1 Polymer Research Centre, Department of Chemical Sciences, Indian Institute of Science Education and Research Kolkata (IISER-K), Mohanpur 741246, Kolkata, India

## Abstract

A novel library of norbornene derived helical copolymer has been synthesized through the coupling of two homopolymers via Molecular Marriage Approach. The helicity is governed by the non-covalent interactions like hydrogen bonding, π-π stacking and the influence of hydrophobic and hydrophilic motifs. The detailed characterization of the copolymer (**Copoly 1**) has been provided and the super structures are confirmed through dynamic light scattering (DLS), scanning electron microscopy (SEM) and transmission electron microscopy (TEM). The observed size of the aggregates was about 200 nm. The density functional theory (DFT) is favorably supported for the formation of proposed structure of **Copoly 1**. Circular dichroism (CD) measurement has confirmed the one handed helical structure of the copolymer. Reservoir capability of this pH responsive polymer (**Copoly 1**) to encapsulate anti-cancer drug doxorubicin (**DOX**) warrants its potential applications in the field of bio-medical sciences.

Amphiphilic block copolymers with the molecular architecture of hydrophobic and hydrophilic moieties are known to self-assemble into various morphologies with well-defined structural order and nanoscale dimensions^1^,^2^,^3^,^4^. Enormous varieties of self-assembled structures such as micelles, vesicles, rod like and sheet like structures, monolayer and bilayer structures are well reported in the literature^5^,^6^,^7^. The self-assembled structures are generally guided by the hydrophobic-hydrophilic interactions, polarity of the solvent, assembly kinetics, pH, temperature, non-covalent interactions like hydrogen bonding and π-π stacking etc. The supramolecular structures formed due to non-covalent interactions has considerable advantage over the structures formed due to step-wise bond formation because of the fewer steps of reactions required compared to the covalent reaction. In addition, the rapid formation of the final product because of the fast and easy process, self-rearrangement of the aggregation to maintain the equilibrium between the components and the final product which will decrease the defect in aggregation compared to the covalent approach^8^. In contrast to other non-covalent interactions, the architectures designed through hydrogen bonding in particular is a subject of interest in the field of supramolecular chemistry due to its directionality and erudite knowledge about the hydrogen bonding donor and acceptor chemistry^9^.

The typical importance of supramolecular nanostructures ascends from the demand of drug and gene delivery vehicles, molecular machines, and modeling bio systems by bio-mimicking. It is well documented in the literature that self-assembled systems promisingly show stimuli responsive nature towards different stimuli such as pH, solvent polarity and temperature^10^,^11^,^12^,^13^,^14^. Amphiphilic block copolymers as the novel drug carrier systems in the field of drug delivery has received enormous interest because of its great drug loading capacity and upturn in the administration of drug in body over a long period of time. Particularly, block copolymer assemblies, especially rod like and patchy aggregates have significantly attracting researcher in the field of drug delivery due to its high loading capacity of hydrophobic objects^15^,^16^,^17^,^18^. Rod like aggregates are having considerably a narrow distribution of diameter compared to the other morphological structures^1^. Stimuli responsive polymers termed as smart drug delivery systems are known to mimic the biological systems by changing the properties^19^. The amphiphilic supramolecular structures of polymers with site-specific functionalities can also preferably accumulate in the cancer cells much more they can do in the normal cells which is a passive drug targeting recognized as enhanced permeability and retention effect (EPR)^20^. In recent years, some efforts have been going on in the field of polymer chemistry to develop the gene delivery more effectively using polymers as the synthetic vector^21^,^22^,^23^. In order to achieve this goal polymer backbone, terminal groups and the side chain can be tuned with binding moieties such as amines accordingly so that it can electrostatically bind the DNA.

Recently, the helical polymers have attained great interest in the field of supramolecular chemistry^24^,^25^. Endo *et al*. and Masuda *et al*. have reported that the incorporation of amino group in the polymer results in the helical structure and established that the origin of helical nature is mainly as a consequence of the intra and inter chain hydrogen bonding between the side group residues in the polymer^26^,^27^,^28^,^29^,^30^,^31^,^32^. Amphiphilic molecules self-assembled into novel architectures that opens a new avenue for creating unique properties of materials and devices^33^,^34^,^35^,^36^,^37^,^38^. Wan and co-workers have reported that the helical structures formed due to hydrogen bonding is highly stabilized, but in most cases these helical structures can be disrupted due to the solvent polarity, temperature, ions, hydrogen bonding agents and alteration in pH^39^. Ring opening metathesis polymerization (ROMP) can govern the molecular architecture by providing polymers with narrow molecular weight distribution; hence this polymerization technique can be used to obtain self-assembled morphologies^40^. Sleiman and co-workers have studied and documented the synthesis of a norbornene containing polymer which has the ability to self-assemble into rod like structures due to the hydrogen bonding interaction between the complementary polymers^41^.

Herein, we report a novel approach to synthesis a norbornene derived helical amphiphilic copolymer **Copoly 1** via the coupling of two homopolymers **Poly 1** and **Poly 2**. The two homopolymers with norbornene backbone are synthesized using ring opening metathesis polymerization assisted by Grubbs’ second generation catalyst^42^,^43^,^44^,^45^,^46^. The **Copoly 1** is having a one handed helical structure which may be due to the inter chain hydrogen bonding between the amine and the carbonyl groups in the polymer side chain. The hydrogen bonding nature of polymer is reflected by the presence of cotton effects in the Circular Dichroism. The stabilized helical structure is having a strong binding efficiency with the anticancer drug doxorubicin (**DOX**) and the genetic material **DNA**. The non-covalent interactions such as hydrogen bonding and π-π stacking between the **Copoly 1** and **DOX**/**DNA** are the driving force of the binding. The dialysis study further explains the stability of the aggregates in the physiological conditions. Detailed characterization of aggregation properties and morphologies are done using scanning electron microscopy (SEM), transmission electron microscopy (TEM), critical aggregation concentration (CAC), and dynamic light scattering (DLS). To best of our knowledge this is the first report for making a new type of helical amphiphilic copolymer based on a simple molecular marriage concept (Fig. 1).

The prime objective of this work was (i) to synthesis a new class of copolymer by simple condensation reaction between two homopolymers and (ii) to demonstrate the stability of **Copoly 1** in physiological conditions, drug carrier capability and doxorubicin (**DOX**) drug delivery from this amphiphilic **Copoly 1.** The **Copoly 1** was prepared by a coupling reaction between **Poly 1** and **Poly 2** in the presence of freshly prepared sodium methoxide.

## Results and Discussion

First of all, the idea of making **Copoly 1** type of copolymer came from the presence of backbone rigidity in the norbornene system. Unlike in acrylates and styrene systems, norbornene system has double bond across the backbone. Further, it is evident from the recent reports that ruthenium based catalyst, particularly with the bulkier monomers, provides predominantly cis-polymers as shown in the Fig. 2. The near-perfect cis,-selectivity of these systems is due to the inversion of the configuration at the metal center that occurs with each propagation step due to the stereogenic metal control. Hence the monomer exclusively approach in an anti fashion to the energetically preferred anti alkylidene^47^,^48^.

**Figure 1:**
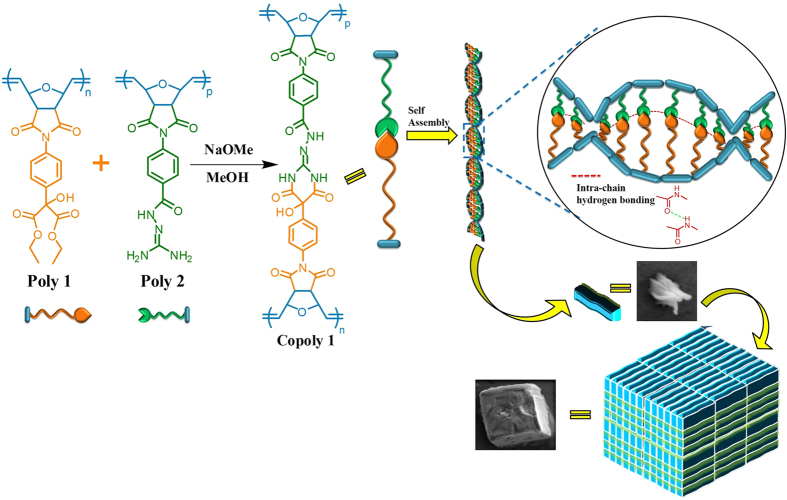
Schematic representation chemical structure of norbornene-derived copolymer’s (Copoly 1) self-assembly.

**Figure 2:**
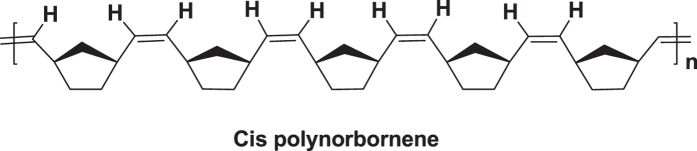
Chemical structure of cis-polymer.

This prompted us to make the **Poly 1** and **Poly 2** with the motivation to make the **Copoly 1**. Since all the functionalities are aligned in the same direction, the reactivity of the **Poly 1** and **Poly 2** is highly favoured as mentioned in the Fig. 1.

The complete synthesis procedure of monomers, homopolymers and **Copoly 1** were discussed in the materials synthesis section (SI Figures S10–S23). The **Copoly 1** was thoroughly characterized using FT-IR, ^1^H and ^13^C NMR spectroscopy and GPC. In FT-IR, the stretching frequency of **Poly 1** at 1709 cm^−1^ for ester carbonyl functional group was absent in **Copoly 1** and a new stretching frequency at 1654 cm^−1^ was appeared for cyclic amide (SI Figure S1). Further it was characterized through ^1^H NMR, the signal at δ 8.5 ppm for amide and δ 5.2 ppm for olefinic protons signals were observed along with aromatic protons signals (SI Figure S2). Finally, the **Copoly 1** sample was injected to GPC, where the molecular weight of the **Copoly 1** was obtained. The peak signal shifted to lower retention time as compared to **Poly 1** and **Poly 2** which confirmed the formation of **Copoly 1**. The observed molecular weight of **Copoly 1** was *M*_n_ = 8200 with a narrow polydispersity index, *Đ* = 1.10 (SI Figure S3, Table 1).

For further support to our claim, density functional theory (DFT) analysis is employed and the results are shown here. All calculations were performed using the B3LYP^49^,^50^,^51^ hybrid density functional^52^,^53^ with 6–31 G(d) basis set as implemented in Gaussian 09^54^.

**Figure 3:**
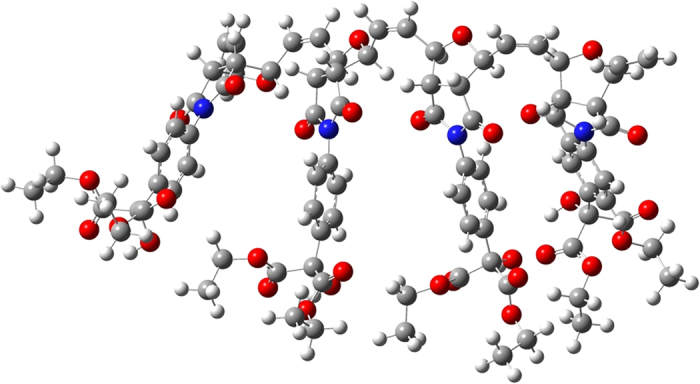
Energy minimized structure of Poly 1 with 4 units.

**Figure 4:**
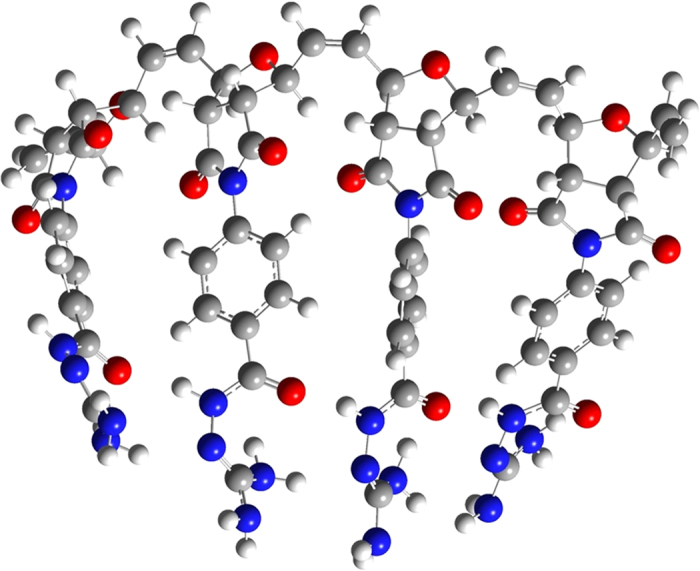
Energy minimized structure of Poly 2 with 4 units.

From the geometry-optimized structures of 4 units of **Poly 1** and **Poly 2**, it is very clear that due to the presence of large number of electronegative atoms in the structure, multiple hydrogen bonding sites are existing in both **Poly 1** and **Poly 2** (Figs 3 and 4). For example, carbonyl carbon of ester groups and –CH_2_ of ethoxy group are involved in H-bonding in **Poly 1**. Similarly, N-H—O=C (amide) and N-H—N=C type of strong H-bondings are involved in **Poly 2**. These types of strong H-bonding keeps the rest of the functionality in a single chain in the same direction. As a result, when first ester group in the **Poly 1** chain approaches the first diamine group in the **Poly 2**, the entire chains of the both the **Poly 1** and **Poly 2** behave like a lock and key type of system, which results in the formation of proposed **Copoly 1**. This also supports the discussion on the absence of starting materials in the product.

**Figure 5:**
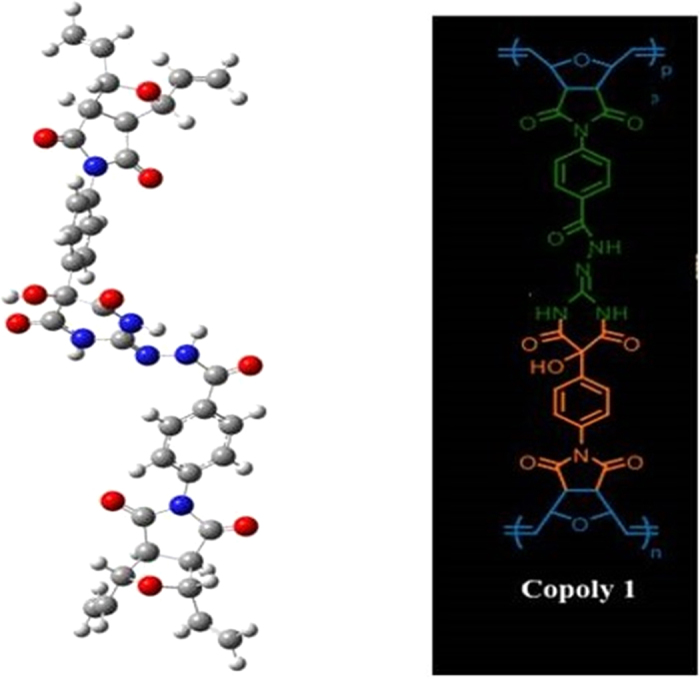
Energy minimized Copoly 1 with 1 unit.

**Figure 6:**
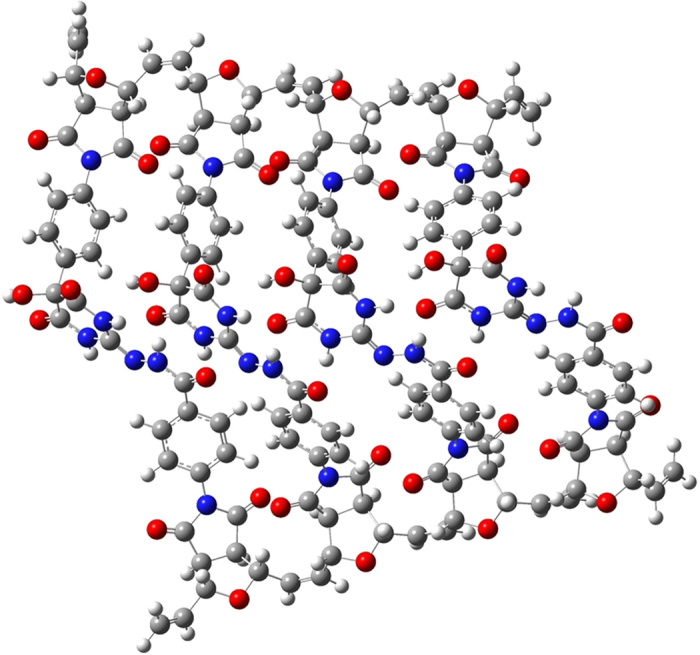
Energy minimized Copoly 1 with 4 units structure.

We have optimized **Copoly 1** with 1 unit and 4 units in the chain, where the chain-ends were capped with =CH_2_ group. From the DFT calculations, it is observed that the energy required to form the structure of **Copoly 1** with 1 unit (Fig. 5) is 22.5 kcal/mol. Based on the calculations of **Copoly 1** with 1 unit, the energy required to make **Copoly 1** of 4 units (Fig. 6) is 90 kcal/mol (22.5 × 4). However, the actual energy observed while making **Copoly 1** with 4 units (Fig. 6) is just 79.4 kcal/mol. The observed energy is significantly low compared to the predicted value. Hence the proposed structure for the formation of **Copoly 1** is favourable.

**Figure 7:**
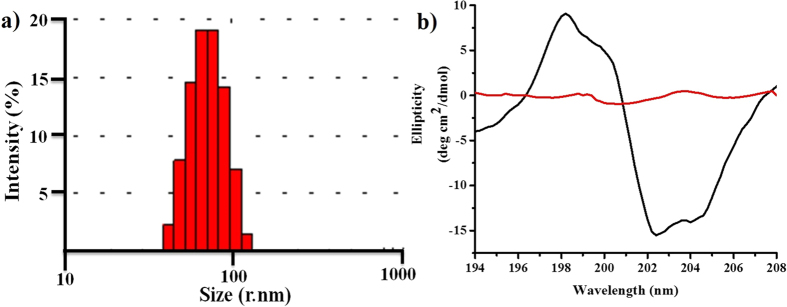
(**a**) DLS measurement of **Copoly 1** in aqueous solution; (**b**) CD analysis of **Copoly 1** before (black) and after (red) heating at 40 °C.

**Figure 8:**
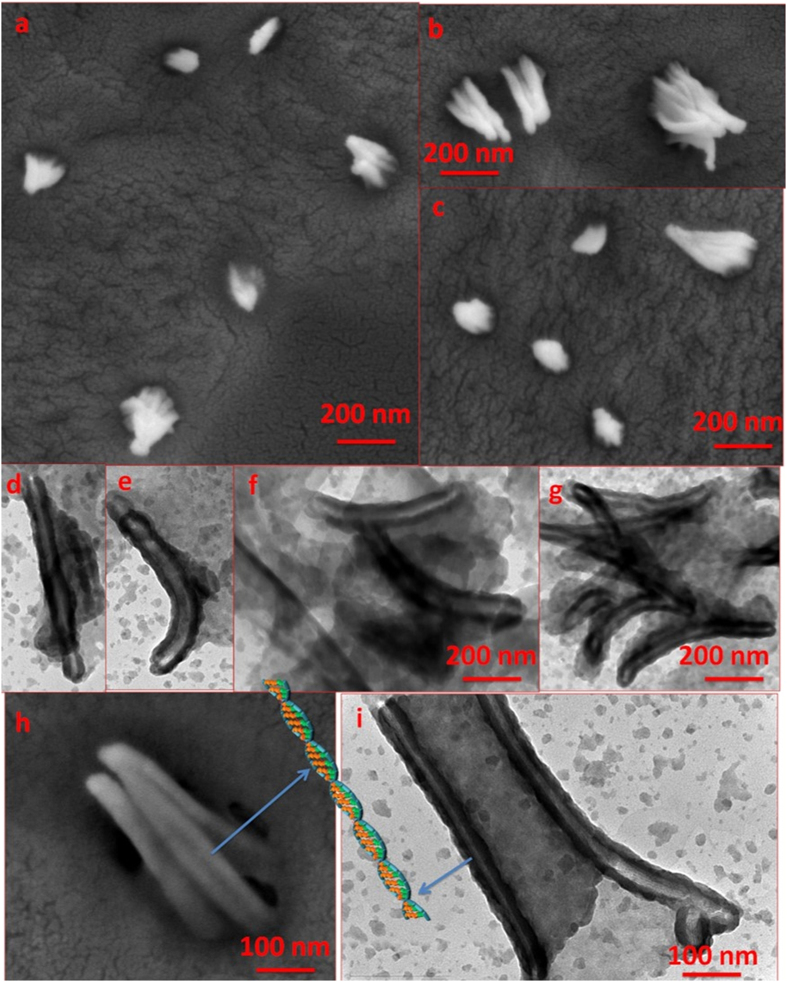
Observed morphology by SEM (**a**–**c** and **h**); and TEM (**d**–**g** and **i**) analysis of **Copoly 1** aggregates dip-casted on a carbon coated copper grid. RuO_4_ was used as the staining agent.

To study the aggregation properties of the **Copoly 1**, Critical aggregation concentration (CAC) was measured using a probe pyrene. The observed CAC was 305 μg/mL (SI Figure S4). After measuring the CAC, the aggregation size of the **Copoly 1** in water solution was measured using dynamic light scattering (DLS). The observed size was 180 ± 0.8 nm, with PDI 0.18 (Fig. 7a). Further, the aggregation properties were explored using Scanning Electron Microscopy (SEM) and Transmission Electron Microscopy (TEM). In SEM, the rod-like superstructures were observed for **Copoly 1**. The observed size of rod-like aggregates was about 200 nm, which had a good agreement with DLS analysis. For TEM analysis, the sample was prepared on a carbon coated copper grid. A series of images were collected and representative images are shown in Fig. 8. Further, the helicity of the **Copoly 1** was studied using circular dichroism spectroscopy (CD), an excellent tool to demonstrate the ellipticity in the aggregates. Strong cotton effects were expected to be found in the polymers having highly ordered structures. The following characteristic peaks were observed; a positive peak at 198.5 nm followed by two negative peaks at 202.5 nm and 204.5 nm (Fig. 7b). The peak at 198.5 nm could be due to the n → π* transition by carbonyl chromophore and the peaks at 202.5 nm as well as 204.5 nm could be responsible for π_1_ → π* transition of amide chromophore. The presence of helical structure was anticipated from the intense CD signals, in particular, the one handed helical nature. It was stabilized by the inter chain hydrogen bonding between the carbonyl and amide groups of the orderly arranged copolymer. It was very interesting to observe the diminishing of the peaks of **Copoly 1** in the CD spectrum with increasing temperature. This influence of heat on the CD spectrum was observed up to 40 °C. This could be due to the disassembly of the hydrogen bonding between the binding sites (Fig. 7b). Recently, White *et al*. reported the hydrophobicity scale, where tryptophan showed largest driving force for the bilayer interfaces. To be precise, the Indole N-H moiety in tryptophan acted as hydrogen bond donar and potentially helped for a cation π-π interaction^55^,^56^. We also explored the interaction of **Copoly 1** to tryptophan to study the denaturation behavior. Tryptophan moieties were introduced into the **Copoly 1** solution; which was expected to play critical hydrogen bonding interactions. Here tryptophan’s absorbane at 268 nm as well as emmission at 340 nm were monitored upon exposure to **Copoly 1**. As the typtophan movement was towards the polar end, the λ_max_ gradually shifted from 340 nm to 350 nm. This interesting 10 nm red shift was observed upon the exposure of **Copoly 1** (SI Figures S5 and S6). This experiment clearly supported our hypothesis that the helicity of the amphiphilic **Copoly 1** was stabilized by the hydrogen bonding between the adjacent moieties.

Further, the DNA binding nature of the **Copoly 1** was explored. The DNA binding experiment was monitored using Circular Dichroism spectroscopy. It was observed that the DNA interacts with the **Copoly 1** (SI Figure S7), The DNA interaction was confirmed by the change in CD spectrum. The interaction between **Copoly 1** and DNA could be due to the hydrogen binding sites such as carbonyl and amide groups present in **Copoly 1** resulted the hydrogen bonding. Also, the bulky groups present in the **Copoly 1** caused the π-π stacking between the DNA and copolymer, which would electrostaticaly make the DNA to bind with the copolymer. The positive band at 220 nm and 285 nm as well as the strong negative band at 248 nm were from the DNA. However, these characeristics bands of DNA started diminishing after the exposer to **Copoly 1**. This binding ability of the DNA with **Copoly 1** prompted us to envision that **Copoly 1** could be furthur explored as nano-vheicle to deliver DNA inside the body.

**Figure 9:**
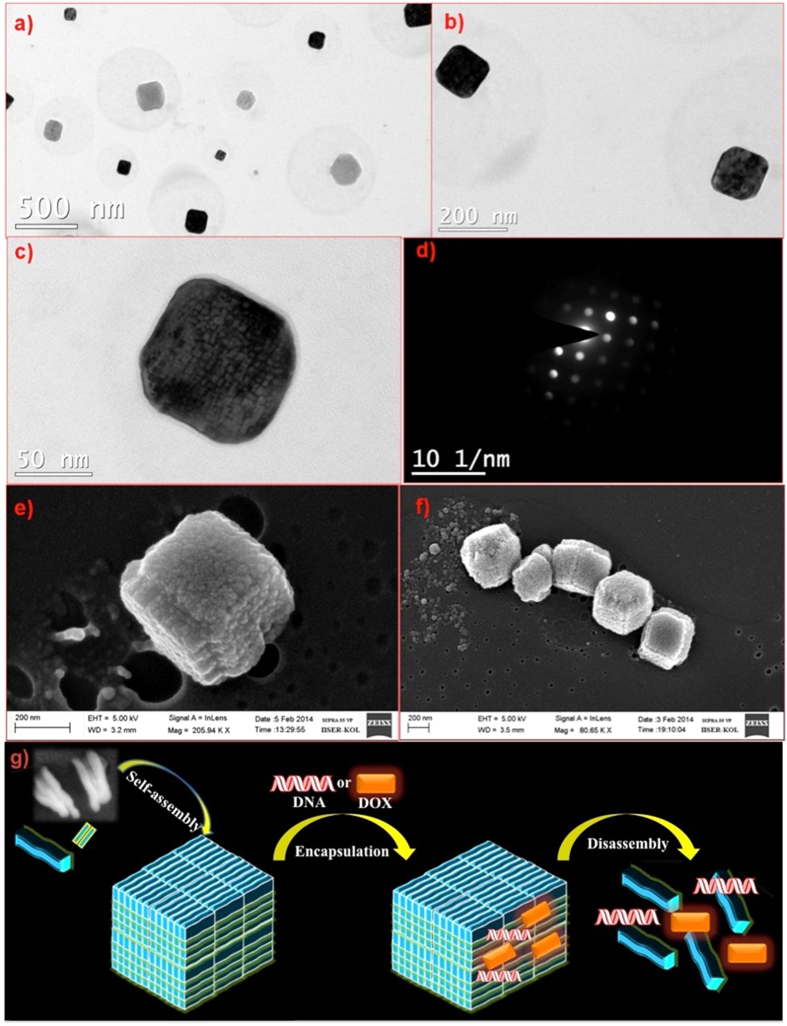
(**a**–**c**) TEM images of cube like assembly of 1 mg of **Copoly 1** in 1 mL water; (**d**) SAED pattern observed for cube in TEM. (**e**,**f**) SEM images. (**g**) Schematic representation for self-assembly of **Copoly 1**; followed by **DOX** and DNA encapsulation and release.

Furthermore, the hierarchical self-assembly of **Copoly 1** with increased concentration was observed by TEM (Fig. 9a–c) and SEM (Fig. 9e–f) analysis. It was evident from the TEM and SEM images that the observed super structures, due to increase in concentration, were cubic in nature. The strong contrast between the dark edges and brighter centers was clearly indicative of this layer-by-layer self-assembly nature. We hypothesized that the polymer chains from **Copoly 1** first self-assembled into rods. With increase in concentration, these rods self-assembled further to form cube-like super structures as shown in Fig. 9g. The nano-cubes were further characterized via a TEM selected area electron diffraction (SAED) studies (Fig. 9d). Not only this analysis confirmed the 3D nature of the nano-cubes, it also pointed to the presence of crystallinity within their external surfaces, as evidenced by the individual aggregates displaying an orientation dependent diffraction contrast. The observed type of pattern shows the primary reflections, in which the bright spots correspond to single cube-like aggregates and the secondary reflection that appeared as less bright spot due to another single crystalline aggregate. With these observations, we proposed that the orientation relationship could be very close to uniform cube like superstructures. Most importantly, the nano-cubes were also of uniform size and shape across the region. DLS result shows size 212 ± 0.68 nm, with PDI 0.16. The size of DOX loaded aggregate is 241 ± 0.9 nm, with PDI 0.27 as well as after disassembly the size is 140 ± 0.8 nm, with PDI 0.15 (SI Figure S9). Schematic representation for self-assembly and drug encapsulation and release process of **Copoly 1** has been demonstrated in Fig. 9g.

Next, to demonstrate the drug carrier capability, doxorubicin (**DOX**) a model anticancer drug in its salt form was used. The encapsulation of hydrophilic **DOX** in **Copoly 1** was done through dialysis method. Both 1.0 mg of **Copoly 1** and 1.0 mg of **DOX** were dissolved in 1.0 mL of water separately and mixed together and stirred for 30 min. Finally the solution mixture was loaded in dialysis tube and dialysed several times with 50 mL of water. This experiment was monitored through fluorescence emission at 552 and 587 nm as an indication of non-encapsulated **DOX** (SI Figure S8). After confirming the complete disappearance of **DOX** emission at 552 nm and 587 nm the solvent was evaporated under reduced pressure. The drug loading efficiency (DLE) of **Copoly 1** was observed about 54% w/w.

**Figure 10:**
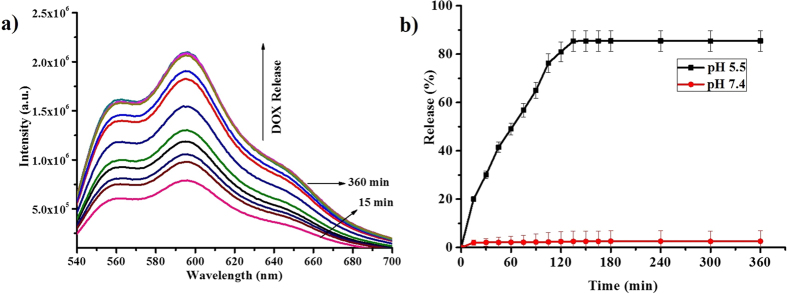
(**a**) Fluorescence spectra for **DOX** release from **Copoly 1**; (**b**) Cumulative **DOX** release from **Copoly 1** at different pH values.

Further, the drug release studies were carried out by dialysis method. The **DOX** encapsulated in **Copoly 1** solution was kept in dialysis tube and dialyzed to 50 mL of buffer solutions of different pH’s 5.5 and 7.4. An aliquot of the sample was removed with different time intervals and the fluorescence emission of **DOX** were measured at 552 nm and 587 nm by exciting the solution at 510 nm as the indication of the **DOX** release from the aggregates. At pH 5.5, 85% of **DOX** release was observed. At pH 7.4, less than 2% of **DOX** release was observed which suggested the stability of **Copoly 1** aggregates in physiological conditions (Fig. 10). The given polymer is not cytotoxic in nature. We have already demonstrated cytotoxicity and cell biocompatibility of the norbornene derived polymeric systems in our previous report^43^.

In summery, here we have successfully synthesized norbornene derived amphiphilic copolymer using a novel molecular marriage approach. The DFT calculations, demonstrated that the energy required to form the **Copoly 1** with 1 unit structure is 22.5 kcal/mol and for 4 units is 90 kcal/mol (22.5 × 4). However, the calculated energy for making **Copoly 1** with 4 units is just 79.4 kcal/mol. Hence the proposed structure for the formation of **Copoly 1** (Molecular Marriage Approach) is favourable. The molecular marriage offers the molecule to form a helical block copolymer. SEM analysis confirmed the rod like aggregates with a size of 200 nm; which was further supported by DLS and TEM. These rods are further self-assemble into cube-like superstructures. The CD spectrum supports the one handed helical nature of the polymeric self-assembly. The tryptophan interaction with **Copoly 1** in CD spectra suggested the hydrogen bonding nature of the polymer. Further, the interaction between **Copoly 1** and DNA were proved by CD spectrum. Alongwith hydrogen bonding, the bulky groups present in the **Copoly 1** were caused π-π stacking between the DNA and copolymer, which promoted DNA electrostaticaly bind with **Copoly 1**. DNA’s characeristics positive bands at 220 nm and 285 nm as well as strong negative band at 248 nm were diminished after exposer to **Copoly 1**. This binding ability of DNA with **Copoly 1** prompted us to envision that **Copoly 1** could be further explored as nano-vheicle to deliver DNA inside the body. Finally, Doxorubicin (**DOX**) a model anti cancer drug was utilized for drug delivery application. This novel **Copoly 1** is proved to be capable of high drug loading efficiency (about 54% w/w). The drug release studies in acidic environment demonstrate the stimuli responsive nature of the novel systems. To best of our knowledge this is the first report for making a new type of helical copolymer based on simple molecular marriage concept.

## Methods

### Materials

All reagents vinyl ethyl ether, second generation Grubbs’ catalyst (G2), tryptophan and deoxyribonucleic acid sodium salt from Escherichia Coli strain B were purchased from Sigma Aldrich and were used as received without further purification. The solvents dichloromethane (DCM) and methanol were dried over calcium hydride (CaH_2_) and used for reactions. The deturated solvents dimethyl sulphoxide-d (DMSO-d_6_) was purchased from Chembridge Isotope Laboratories.

### Materials Synthesis

#### Synthesis of 1

Exo - oxabicylo- [2.2.1] hept-5-ene-2, 3 - dicarboxylic anhydride (**1**) was prepared as per literature procedure. In a two neck round bottom flask, 10 g (102 mmol) of maleic anhydride was dissolved in 80 mL of toluene. After stirring for 30 min, it was filtered. To the filterate, 8.32 g (122.4 mmol) of furan was added. This solution was stirred for 48 h at room temperature. White colour solid was precipitated out from the solution. Then the precipitate was filtered, and finally washed with cold toluene (96% yield). ^1^H NMR (500 MHz, CDCl_3_): δ 6.56 (s, 2H), 5.44 (s, 2H), 3.17(s, 2H); ^13^C NMR (125 MHz, dmso-d_6_): δ 171.54, 137.20, 81.63, 49.08; MS (ESI) calculated for C_8_H_6_O_4_ (M^+^+H) 166.027, observed 166.

#### Synthesis of 2

5 g (30.12 mmol) of exo - oxabicylo- [2.2.1] hept-5-ene-2, 3 - dicarboxylic anhydride (**1**) and 2.8 g (30.12 mmol) of aniline was dissolved in 50 ml of methanol. The reaction mixture was stirred at 56 °C for 72hr. This reaction mixture concentrated to the pasty mass and this pasty mass was dissolved in dichloromethane (50 ml), and washed with water (3 × 100 ml). The organic layer was dried over NaSO_4_ and concentrated under vacuum to yield molecule **2** as a brownish solid (74% yield). ^1^H NMR (500 MHz, dmso-d_6_): δ 7.29–7.6 (d, 4H), 7.19 (t, 1H) 6.5 (s, 2H), 5.2 (s, 2H), 3.0 (s, 2H); ^13^C NMR (500 MHz, DMSO-d_6_): δ 172.01, 137.31, 131.90, 128.01, 82.10, 49.53; MS (ESI) calculated for C_14_H_11_NO_3_ (M^+^+H) 241.07, observed 241.62.

#### Synthesis of 3

200 mg of **2** was dissolved in 5 ml of dichloromethane in a two necked round bottom flask fitted with CaCl_2_ guard tube and the flask was kept in ice bath to avoid vigorous reaction. Diethyl oxomalonate (1 mL) was added via syringe followed by drop wise addition of 0.9 ml of stannous chloride. The reaction mixture was stirred at room temperature for 6 hr. The reaction mass was concentrated under vacuum to yield brownish solid (65% yield). ^1^H NMR (500 MHz, dmso-d_6_): δ 7.49 (s, 2H), 7.32 (s, 1H), 7.21 (s, 2H), 6.61 (s, 2H), 5.21 (s, 2H), 4.18 (m, 4H), 3.14 (s, 2H), 1.18(m, 6H); ^13^C NMR (500 MHz, DMSO-d_6_): δ 176.04, 165.3, 137, 132.5, 129.1, 129, 128.1, 128, 92.3, 81.2, 61.7, 48.03, 14.1. MS (ESI) calculated for C_21_H_21_NO_8_ (M^+^+H) 415.12, observed 416.

#### Synthesis of 4

Exo - oxabicylo- [2.2.1] hept-5-ene-2, 3 - dicarboxylic anhydride (**1**), 1.914 g (11.5 mmol) was dissolved in acetone (35 mL) and heated until it became clear solution. To this solution, 1.605 g (11.5 mmol) of p- amino benzoic acid was added and the reaction mixture was allowed to stir at room temperature for 30 min. The solid was filtered and dried under vacuum. The dried precipitate was then dissolved in 30 mL of dimethyl formamide and heated to 50 °C. Acetic anhydride 15 mL (158.97 mmol) and sodium acetate 0.635 g (7.743 mmol) were added to the solution under stirring. The reaction mixture was allowed to stir for 3 h at 55 °C. After 3 h, the reaction mixture was poured into 500 mL of water and acidified by addition of 5 mL of concentrated HCl. White colour solid was precipitated out from the solution immediately. The precipitate was filtered, washed with water several times and dried under the vacuum (80% yield). ^1^H NMR (DMSO-d_6_, 400 MHZ): δ 13.03 (bs, 1H), 8.0–8.2 (m, 2H), 7.4–7.5 (m, 2H), 6.6 (s, 2H), 5.34 (s, 2H), 3.1 (s, 2H). ^13^C NMR (DMSO-d_6_, 400 MHz): δ 175.43, 166.59, 136.65, 135.78, 130.0, 126.79, 80.86, 47.58. IR (KBr, cm^−1^): 3236, 2635,2073,1954,1826, 1780, 1729, 1698, 1607, 1515, 1418, 1218, 1144, 1125, 1020, 975, 950, 912, 883, 878, 804, 726, 672, 633, 598, 541, 521. MS (ESI) calculated for C_8_H_10_O_2_Na [M^+^+H] 285.05, observed 284.91.

#### Synthesis of 5

0.80 g (4.21 mmol) of N-(3-dimethylaminopropyl)-N′-ethylcarbodiimide hydrochloride (EDC.HCl) and 1-hydroxybenzotriazole (HOBt) 0.64 g (4.21 mmol) were added to a solution of **4** (1 g, 3.50 mmol) in dimethyl formamide (10 mL). The reaction mixture was allowed to stir for 15 h at room temperature. Then the reaction mixture was cooled at 0–5 °C. To this, tertiary butyl carbazate 0.46 g (3.50 mmol) in dimethyl formamide was added. Then the reaction mixture was stirred for another 30 min at 0–5 °C. After that 30 mL of ethyl acetate followed by 30 mL water were added to the reaction mixture.Then the organic layer was washed with water followed by sodium bicarbonate. Finally, the organic layer was washed with brine solution and then concentrated under vacuum to yield a white colour solid (88% yield). ^1^H NMR (500 MHz, dmso-d_6_): δ10.92 (s, 1H), 8.9 (s, 1H) 7.97 (d, 2H), 7.42(d, 2H), 6.66 (s, 2H), 5.26 (s, 2H), 1.21(s, 9H); ^13^C NMR (125 MHz, dmso-d_6_): δ 176.01, 166, 155.2, 137, 132.21, 129.02, 127.1, 81.7, 48.61, 27.2; MS (ESI) calculated for C_20_H_21_N_3_O_6_ (M^+^+H) 399.397, observed 399.18.

#### Synthesis of 6

50 mg of molecule **5** was taken into a two neck round bottom flask. It was dissolved in 1 mL of dichloromethane. The reaction mixture was kept under nitrogen atmosphere. Into another flask, 1.06 mg (100 mol %) of second generation Grubbs’ catalyst was added. Both the flasks were degassed for three times by freeze-pump-thaw cycles. Finally the molecule 5 solution was transferred to the flask containing the catalyst via a cannula. The reaction mixture was allowed to stirr at room temperature until the polymerization was complete (12 hr). An aliquot of sample was taken for GPC analysis. Finally the remaining product was quenched with ethyl vinyl ether (0.2 mL). It was precipitated from pentane, dissolved it again in THF, passed it through neutral alumina to remove the catalyst and precipitated again from pentane to get a pure homopolymer **6**. Gel permeation chromatography was done in tetrahydrofuran (flow rate = 1 ml/min). The molecular weight of polymer was measured using polystyrene standards. (M_n_ = 4300, PDI = 1.1).

#### Synthetic procedure of **poly 1**

For the synthesis of **poly 1**, 50 mg of **3** was taken into a separate Schlenk flask. It was dissolved in methanol and anhydrous dichloromethane (1:9 v/v%) solvent mixture. The reaction mixture was kept under an atmosphere of nitrogen. Into another Schlenk flask, 1.06 mg (100 mol%) of second generation Grubbs’ catalyst was added, flushed with nitrogen, and dissolved in minimum amount of anhydrous dichloromethane. Both flasks were degassed for three times by freeze-pump-thaw cycles. Then the molecule **3** solution was transferred to the flask containing the catalyst via a cannula. The reaction was allowed to stirr at room temperature until the polymerization was complete (24 hr). An aliquot was taken for GPC analysis. Finally the remaining product was quenched with ethyl vinyl ether (0.2 mL). It was precipitated from pentane, dissolved it again in THF, passed it through neutral alumina to remove the catalyst and precipitated again from pentane to get a pure homopolymer. Gel permeation chromatography was done in tetrahydrofuran (flow rate = 1 ml/min). The molecular weight of polymer was measured using polystyrene standards. (*M*_*n*_ = 4300, PDI = 1.11).

#### Synthetic procedure of **poly 2**

A known amount of molecule **6** (500 mg) was dissolved in 5 mL of dichloromethane. To the rection mixture tri fluoroacetic acid 5 mL was added and stirred for 1 hr at room temperature. Reaction mixture concentrated to pasty mass, and charged diethyl ether resultant product was collected by suction filtration, washed with 10 mL diethyl ether and dried at 40 °C under vacuum. This solid product was again dissolved in methanol. Finally Urea was added and stirred reaction mixture for 24 hr at 45 °C. The **poly 2** was isolated by precipitation with diethyl ether. The molecular weight of polymer was measured using polystyrene standards. (*M*_n_ = 4700, PDI = 1.07).

#### Synthetic procedure of **copoly 1**

A known amount of **poly 1** and **poly 2** (1:1) was weighed into a two neck round bottom flask, placed under an atmosphere of nitrogen and dissolved in anhydrous methanol. To the reaction mixture freshly prepared sodium methoxide was added and stirred for 24 hr at room temperature. The copolymer was isolated by precipitation with diethyl ether. The molecular weight of polymer was measured using polystyrene standards. (M_n_ = 8200, PDI = 1.10).

#### Critical Aggregation Concentration Study for **Copoly 1**

The concentration of pyrene was maintained at 0.2 μM, while the concentration of **Copoly 1** was changed from 10 to 1500 μg/mL. The excitation wavelength was set at 339 nm, and the emission intensities were measured at 371, 382, and 391 nm. The ratio of the intensity of the first and third peaks (391/371) in the emission spectrum of pyrene was indicative of the polarity of its microenvironment. The relative emission intensities of 391/371 nm were varied as a function of polymer **Copoly 1** concentration. The CAC was determined from the **Copoly 1** concentration value at which the relative fluorescence intensity ratio began to change. The observed CAC was 305 μg /mL.

#### Doxorubicin (DOX) encapsulation/release studies

For the encapsulation studies, 1 mg of **copoly 1** and 1 mg of doxorubicin (**DOX**) in its salt form were dissolved in 1 mL of water separately. After thorough mixing of these two solutions for 30 mins, the mixture was loaded in a dialysis tube (3,500; Dalton cut-off) and dialyzed against high-pure water. The complete removal of **DOX** in the peripheral of the **copoly 1** was confirmed by following the DOX emission at 552 nm and 587 nm. The dialysis was done until the complete disappearance of DOX emission from the dialyzed solution. Similar procedure was followed for other solvent systems.

The drug loading efficiency (DLE) was calculated according to the following formula:


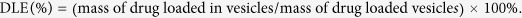


After the drug encapsulation process, the drug release studies were carried out at room temperature by dialysis method. The **DOX** encapsulated in **Copoly 1** solution was kept in dialysis tube (3500; Dalton cut-off) and dialyzed to 50 mL buffer solution of different pH’s 5.5 and 7.4. An aliquot of the sample was removed with different time intervals and the fluorescence emission of **DOX** were measured at 552 nm and 587 nm by exciting the solution at 510 nm as the indication of the release from the aggregates. This procedure was repeated for every 15 min by maintaining the constant volume of the solution.

#### DNA binding study

1 mg of DNA was dissolved in 10 mL water and in another vial 1 mg of **Copoly 1** was dissolved in 10 mL water. The circular dichroism spectrums for both the solutions were measured. Finally both the solution mixed together (1:1 vol) and measured the circular dichroism spectra.

#### Tryptophan encapsulation Study

A stock solution was prepared from 1 mg of tryptophan in 1 mL water and 1 mg of **Copoly 1** in 1 mL water. Pipette out 10 μL of tryptophan solution and further diluted to 3 mL water and measured its absorbance at 268 nm as well as emission at 340 nm. To this solution 10 μL of **Copoly 1** solution was added and measured the absorbance at 268 nm and emission at 340 nm. It was observed that the decrease in absorbance as well as shifts in emission clearly demonstrate the encapsulation of tryptophan.

### Materials Characterizations

#### Gel Permeation Chromatography (GPC)

Molecular weights and PDIs were measured by Waters gel permeation chromatography in THF relative to polymethylmethacrylate (PMMA) as well as polystyrene (PS) standards on systems equipped with Waters Model 515 HPLC pump and Waters Model 2414 Refractive Index Detector at 35 °C with a flow rate of 1 mL/min. HRMS analyses were performed with Q-TOF YA263 high resolution (Waters Corporation) instruments by +ve mode electrospray ionization.

#### Fluorometry

Fluorescence emission spectra were recorded on a Fluorescence spectrometer (Horiba Jobin Yvon, Fluromax-3, Xe-150 W, 250–900 nm).

#### Nuclear Magnetic Resonance (NMR)

The ^1^H NMR spectroscopy was carried out on a Bruker 500 MHz spectrometer using DMSO-d_6_, D_2_O and CDCl_3_ as a solvent. ^1^H NMR spectra of solutions in DMSO-d_6_, D_2_O and CDCl_3_ were calibrated to tetramethylsilane as internal standard (δH 0.00).

#### Fourier Transform Infra Red (FT-IR)

FT-IR spectra were obtained on FT-IR Perkin-Elmer spectrometer at a nominal resolution of 2 cm^−1^.

#### Ultra Violet (UV) Spectroscopy

UV-visible absorption measurements were carried out on U-4100 spectrophotometer HITACHI UV-vis spectrometer, with a scan rate of 500 nm/min.

#### Dynamic Light Scattering (DLS)

Particle size of polymer was measured by dynamic light scattering (DLS), using a Malvern Zetasizer Nano equipped with a 4.0 mW He-Ne laser operating at λ = 633 nm. All samples were measured in aqueous as well as methanol at room temperature and a scattering angle of 173°.

#### Transmission Electron Microscopy (TEM)

Low resolution transmission electron microscopy (TEM) was performed on a JEOL 200 CX microscope. TEM grids were purchased from Ted Pella, Inc. and consisted of 3–4 nm amorphous carbon film supported on a 400-mesh copper grid.

#### Scanning Electron Microscopy (SEM)

High resolution SEM was performed on a Zeiss microscope; SUPRA 55VP-Field Emission Scanning Electron Microscope. High performance variable pressure FE-SEM with patented GEMINI column technology. Schottky type field emitter system, single condenser with crossover-free beam path. Resolution: 1.0 nm at 15 kV; 1.6 nm at 1 kV high vacuum mode. 2.0 nm at 30 kV at variable pressure mode.

## Additional Information

**How to cite this article:** Mane, S. R. *et al*. Synthesis of Norbornene Derived Helical Copolymer by Simple Molecular Marriage Approach to Produce Smart Nanocarrier. *Sci. Rep.* **7**, 44857; doi: 10.1038/srep44857 (2017).

**Publisher's note:** Springer Nature remains neutral with regard to jurisdictional claims in published maps and institutional affiliations.

## Supplementary Material

Supplementary Information

## Figures and Tables

**Table 1:** Table for GPC analysis for **Poly 1, Poly 2** and **Copoly 1–3.**

Entry	Homopolymer				Copolymer		
	Poly 1	*M*_n_^b^	Poly 2	*M*_n_^b^	*M*_n_^a^	*M*_n_^b^	PDI^c^
Copoly 1	1a	4300	2a	4700	8100	8200	1.10
Copoly 2	1b	3300	2b	3400	6000	6100	1.08
Copoly 3	1c	2100	2c	2300	4000	4200	1.12

^a^*M*_*n*_ Target.

^b^*M*_*n*_ by GPC using polystyrene as a standard.

^c^Ratio of (*M*_w_/*M*_n_).
