# Supplementary material for: Synthesis of Norbornene Derived Helical Copolymer by Simple Molecular Marriage Approach to Produce Smart Nanocarrier
Source: Sci Rep. 2017 Mar 22;7:44857. doi: 10.1038/srep44857 (PMC5361182; doi:10.1038/srep44857)
Supplement: Supplementary Information [file srep44857-s1.doc]

Synthesis of Norbornene Derived Helical Copolymer by Simple Molecular Marriage Approach to Produce Smart Nanocarrier

Shivshankar R. Mane,^1^ Ashlin Sathyan^1^ and Raja Shunmugam^1^*

^1^Polymer Research Centre, Department of Chemical Sciences, Indian Institute of Science Education and Research Kolkata (IISER-K), Mohanpur 741246, Kolkata, India.

Email: sraja@iiserkol.ac.in

**Synthesis Scheme S1:**

**Additional Figure's:**


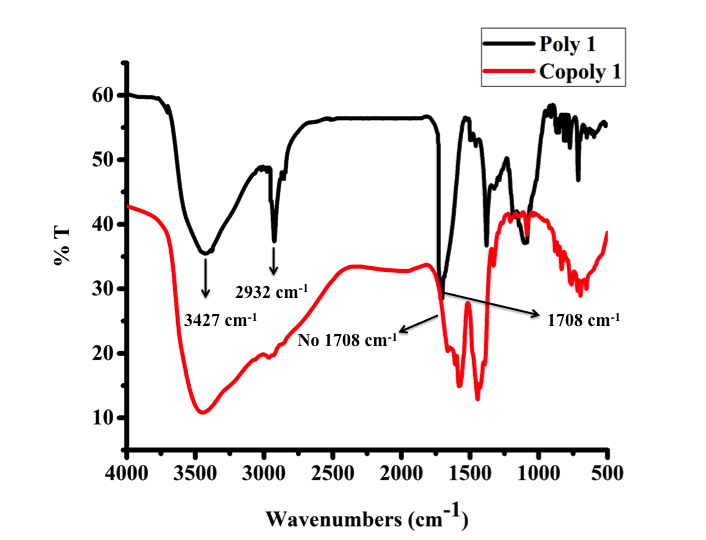


**Fig S1:** FT-IR spectra for **Poly 1** and **Copoly 1**.


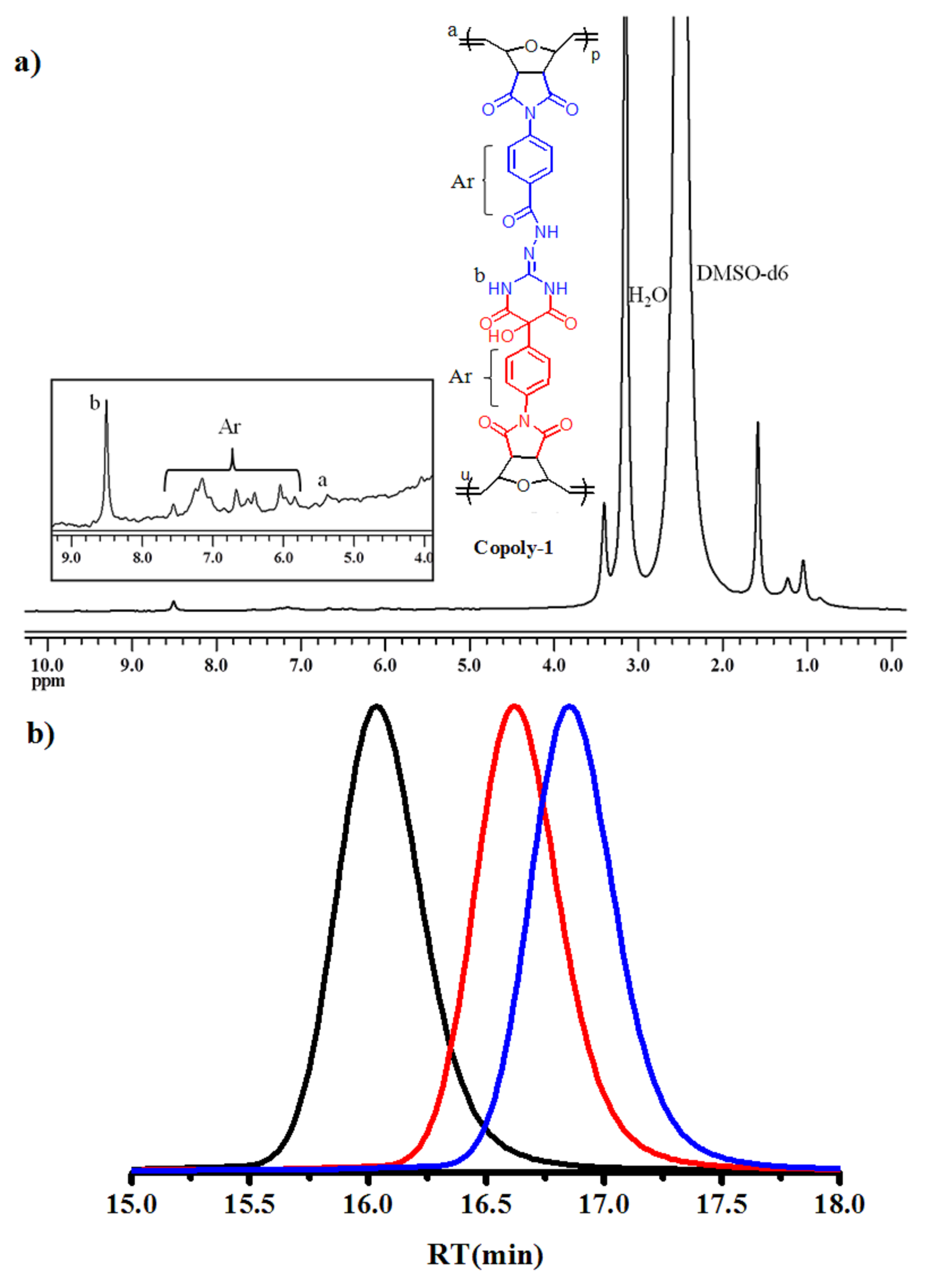


**Fig S2:**  ^1^H NMR spectra for **Copoly 1** in DMSO-*d*_6_.

**
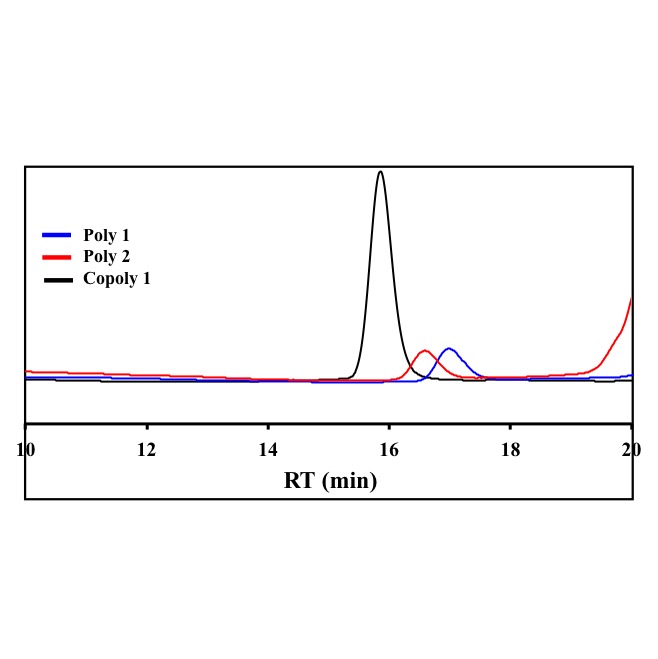
**

**Fig S3:** GPC traces for **Poly 1**(*M_n_*= 4300,PDI=1.11); **Poly 2** (*M_n_*= 4700,PDI=1.07); and  **Copoly 1** (*M_n_*= 8200,PDI=1.1).

**
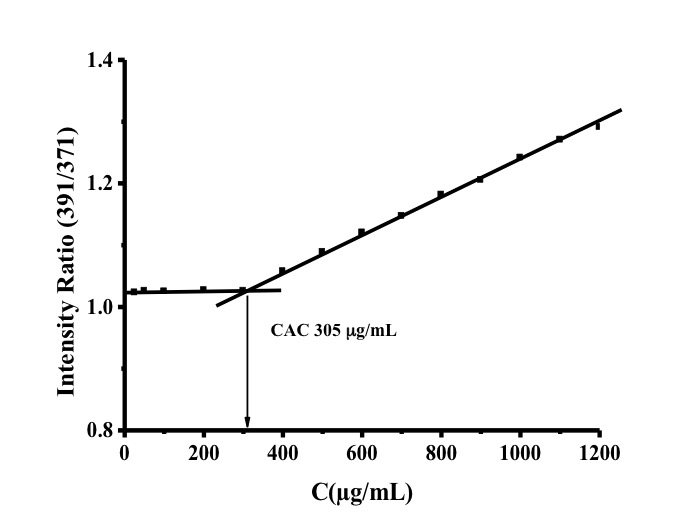
**

**Fig S4:** CAC determination for **Copoly 1.**

**Fig S5:**  UV spectra for tryptophan encapsulation in **Copoly 1.**

**Fig S6:**  Fluorescence spectra for tryptophan encapsulation in **Copoly 1.**

**
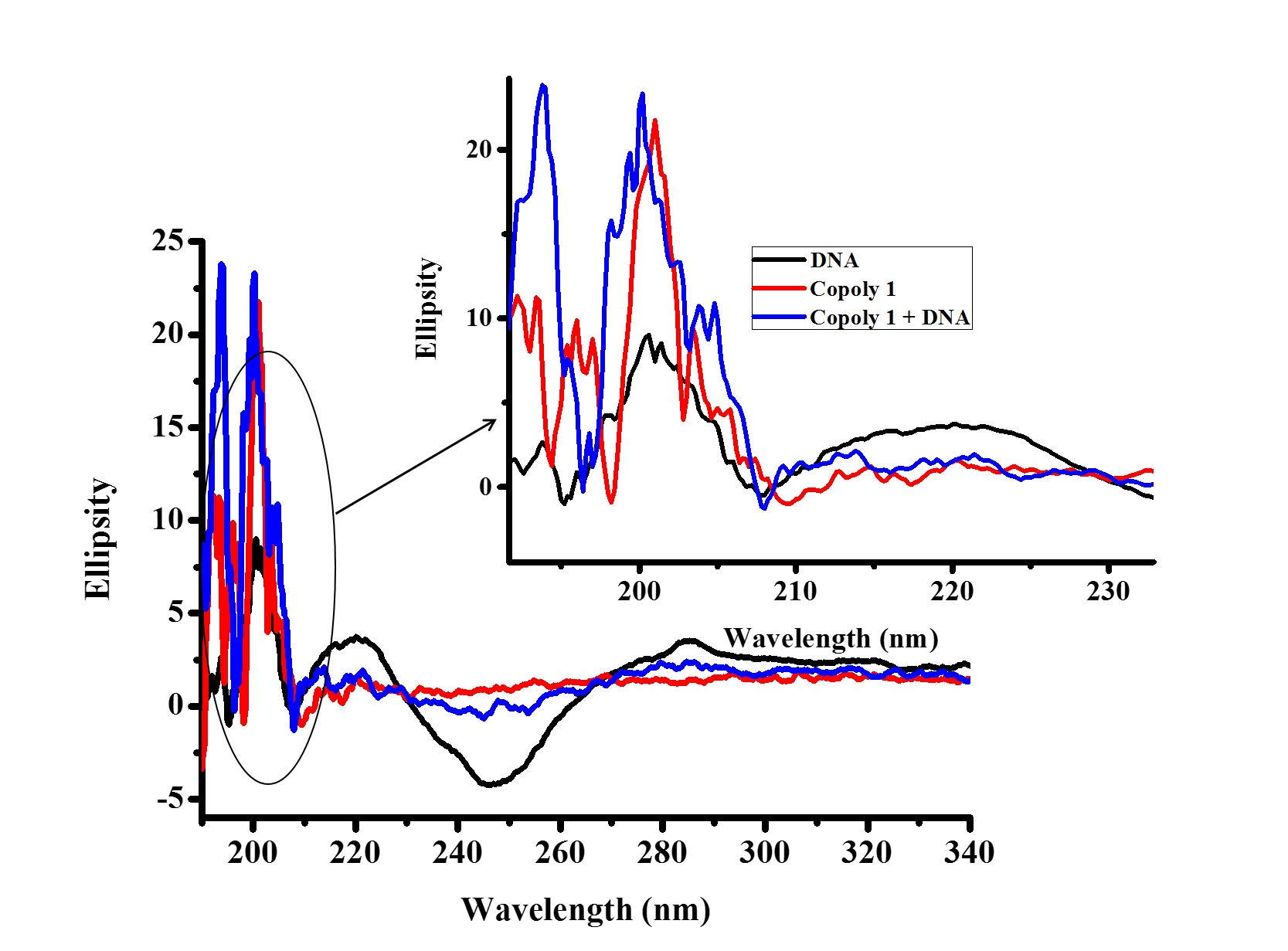
**

**Fig S7:**  CD spectra for DNA encapsulation in **Copoly 1** (red), **DNA** (black), **Copoly 1 + DNA** (blue).

**
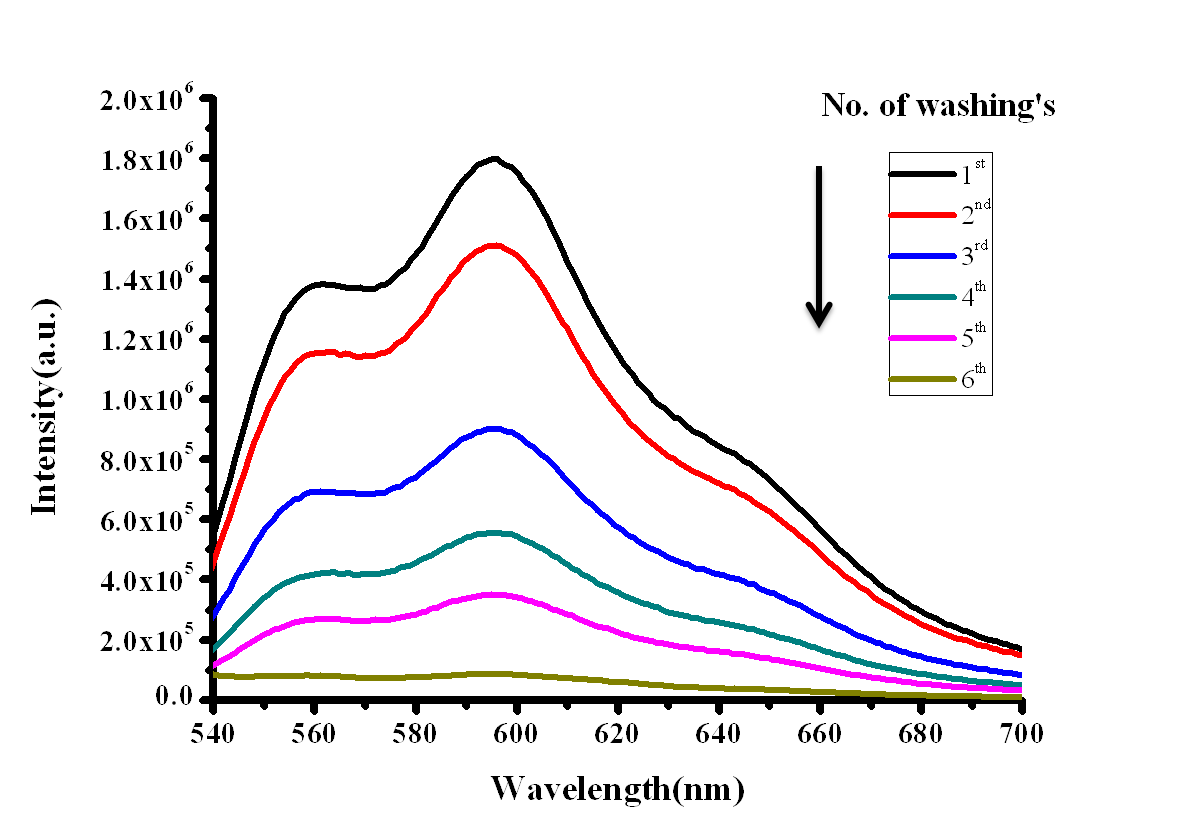
**

**Fig S8:** Fluorescence spectra for **DOX** encapsulation in **Copoly 1** by dialysis method.


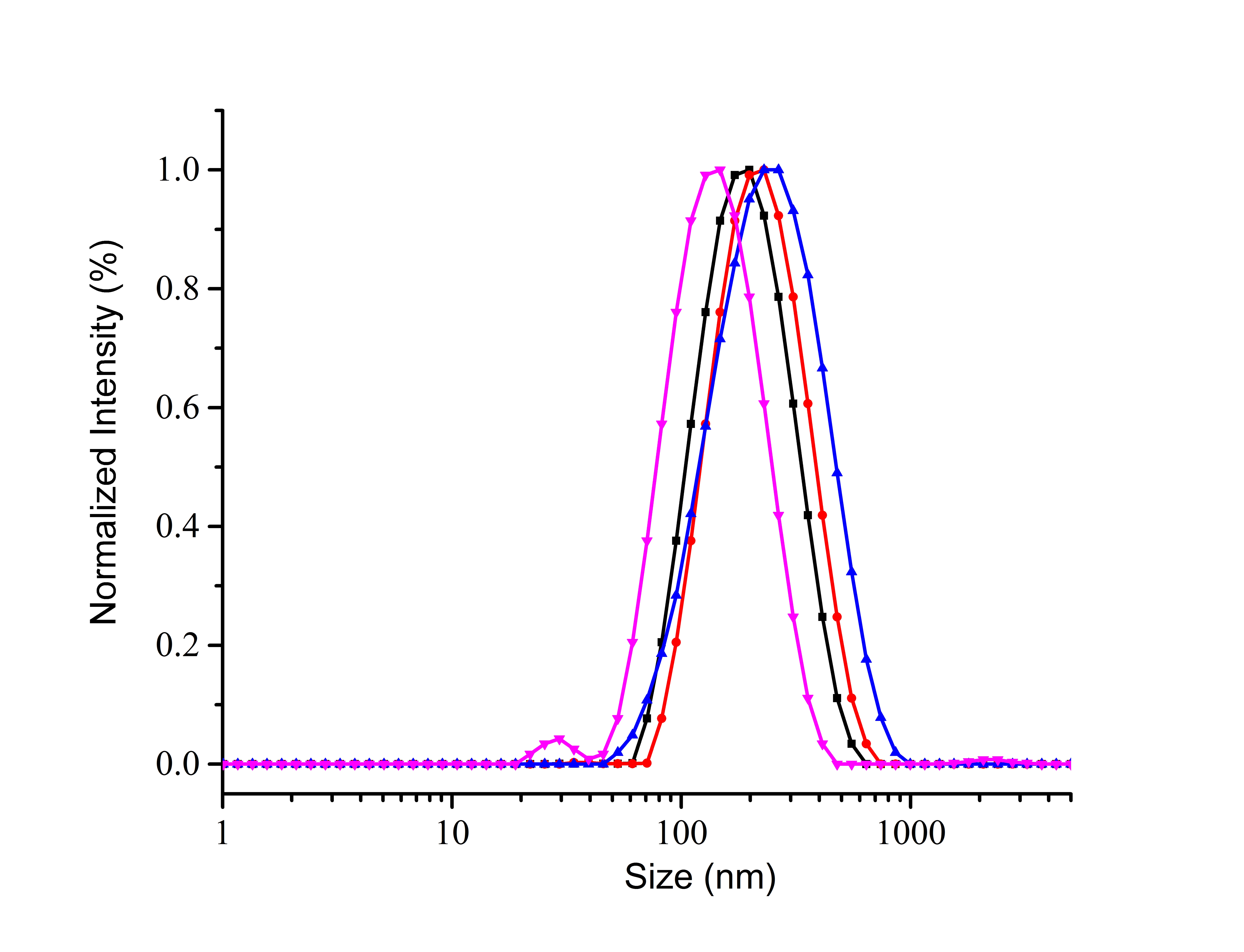


**Fig S9:** Size measurement by DLS**: Copoly 1- rods** (black)**, Copoly 1-cube** (red), **DOX** encapsulation in **Copoly 1** (blue), **Copoly 1** disassembly **(**cyan).

**
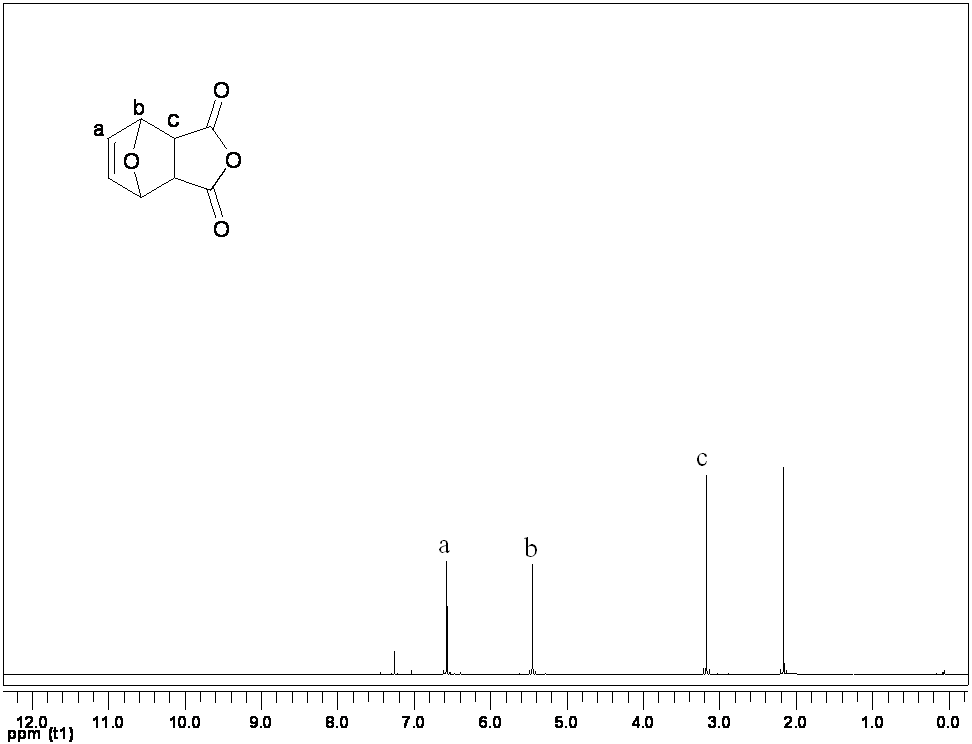
**

**Fig S10**: ^1^H NMR of compound **1** in CDCl_3_.

**
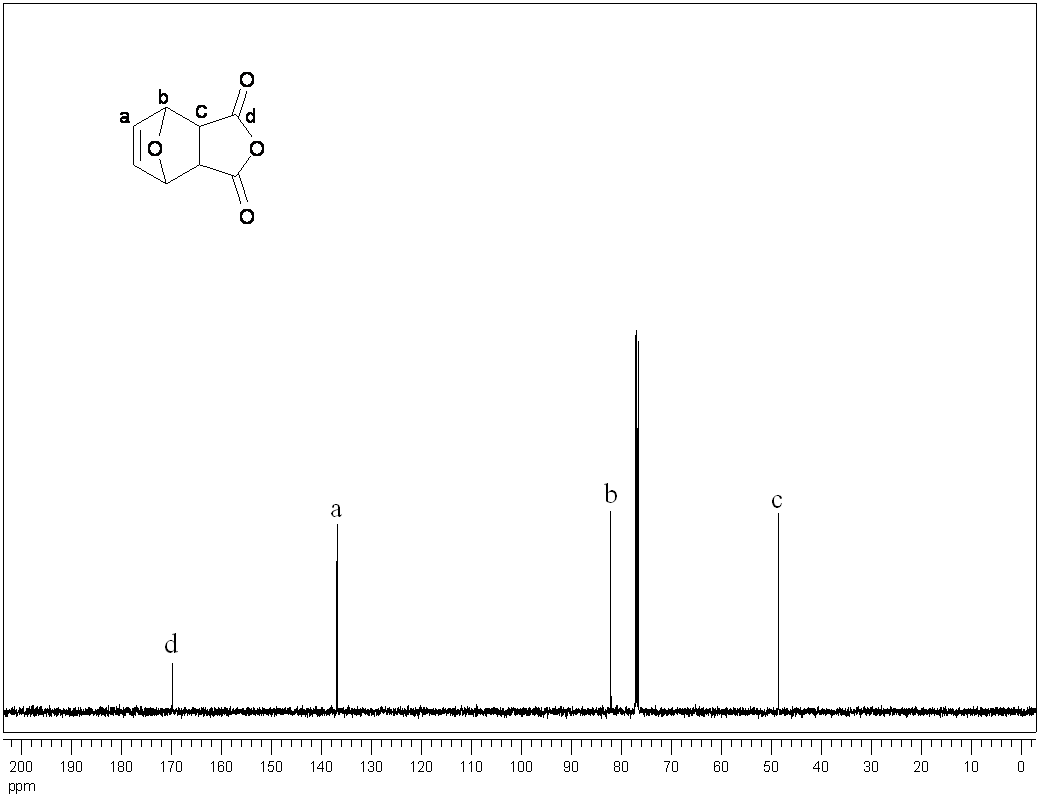
**

**Fig S11:** ^13^C NMR of compound **1** in CDCl_3_.


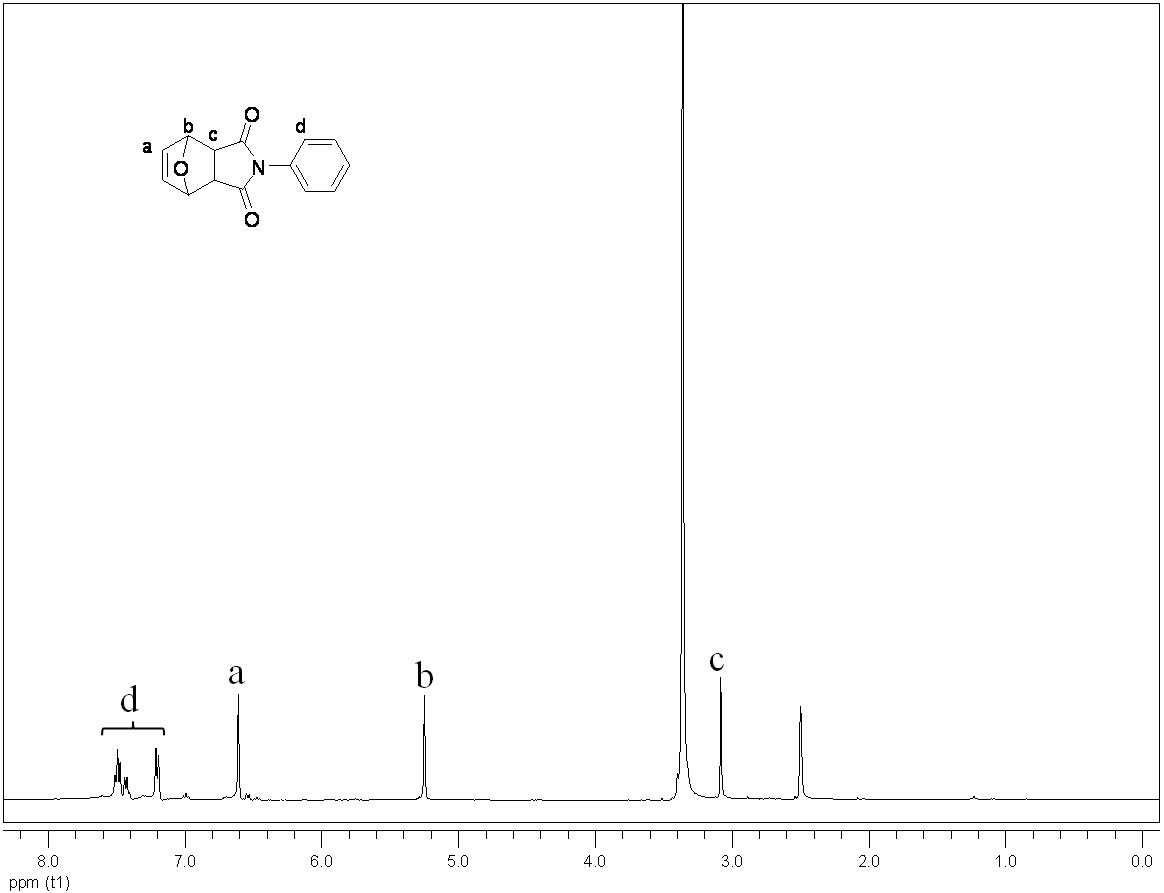


**Fig S12:**  ^1^H NMR of compound **2** in DMSO-d_6_.

**
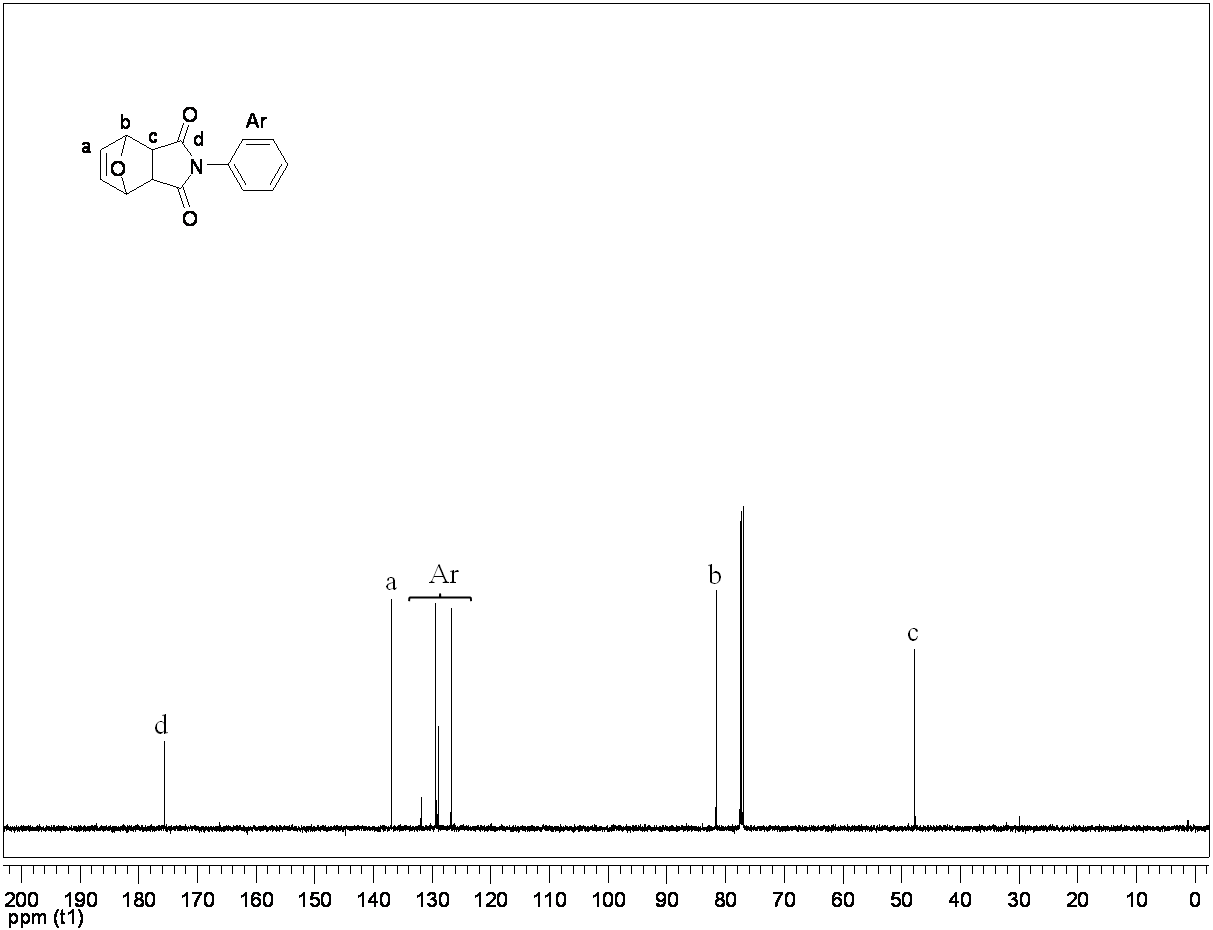
**

**Fig S13:** ^13^C NMR of compound **2** in CDCl_3_.


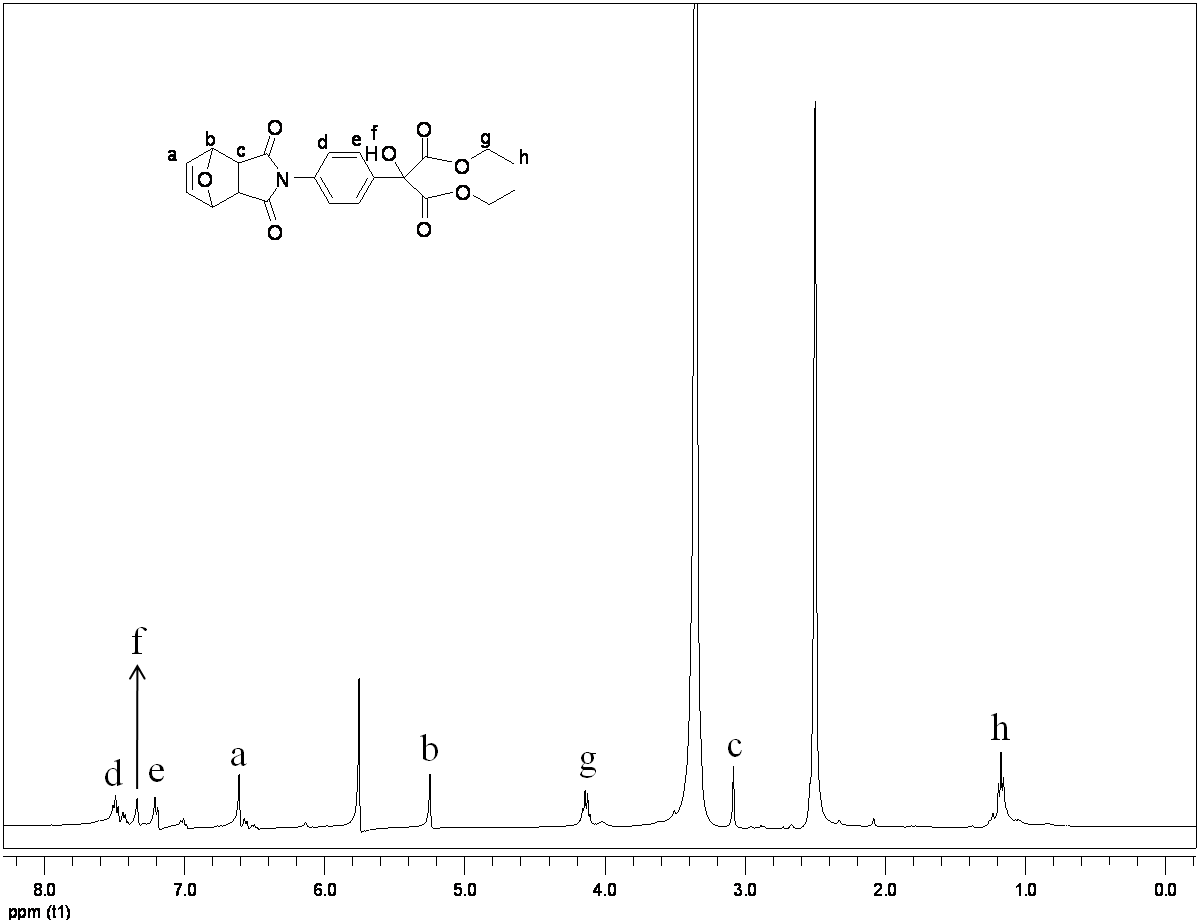


**Fig S14:** ^1^H NMR spectra for molecule **3** in DMSO-d6**.**

**
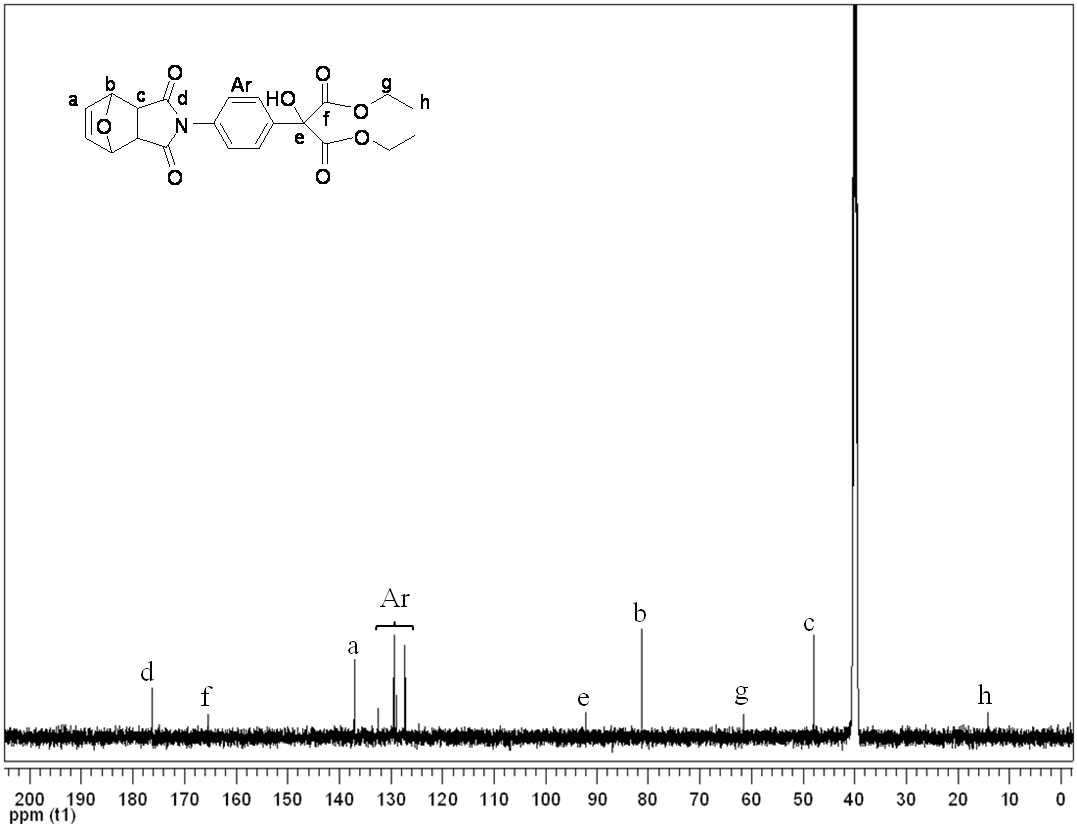
**

**Fig S15:** ^13^C NMR spectra for molecule **3** in DMSO-d6.


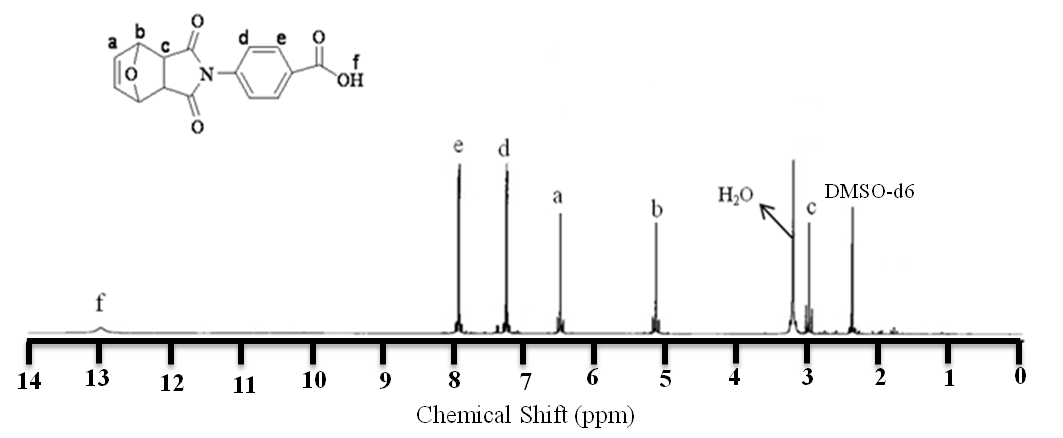


**Fig S16**: ^1^H NMR spectrum of compound **4** in DMSO-d_6_.


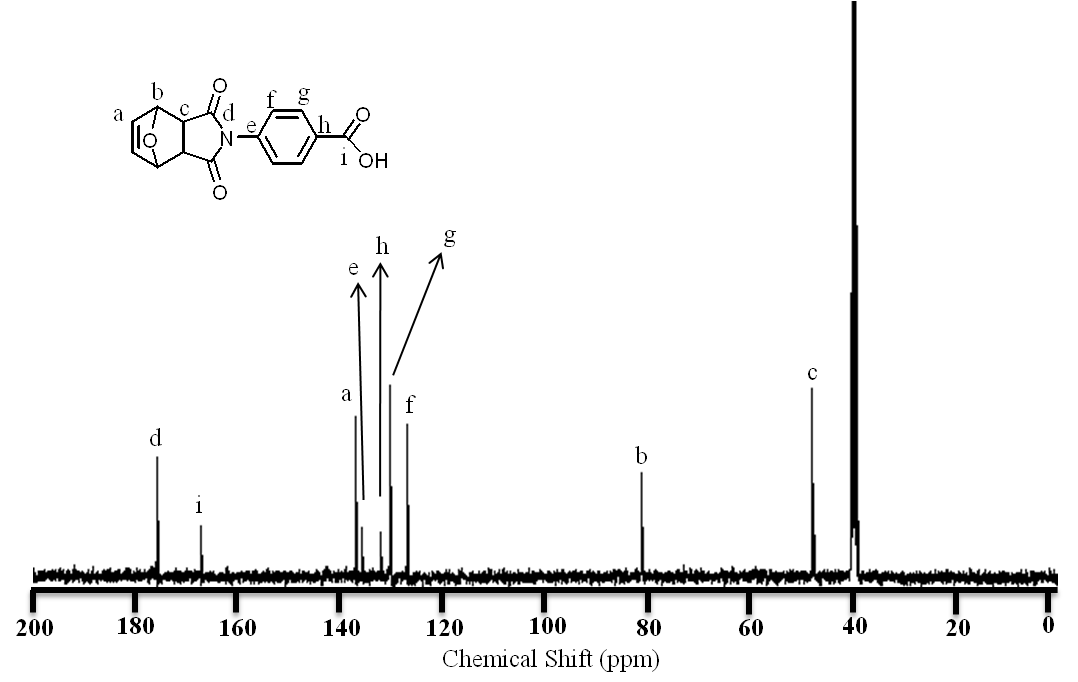


**Fig S17**: ^13^C NMR spectrum of compound **4** in DMSO-d_6_.

**
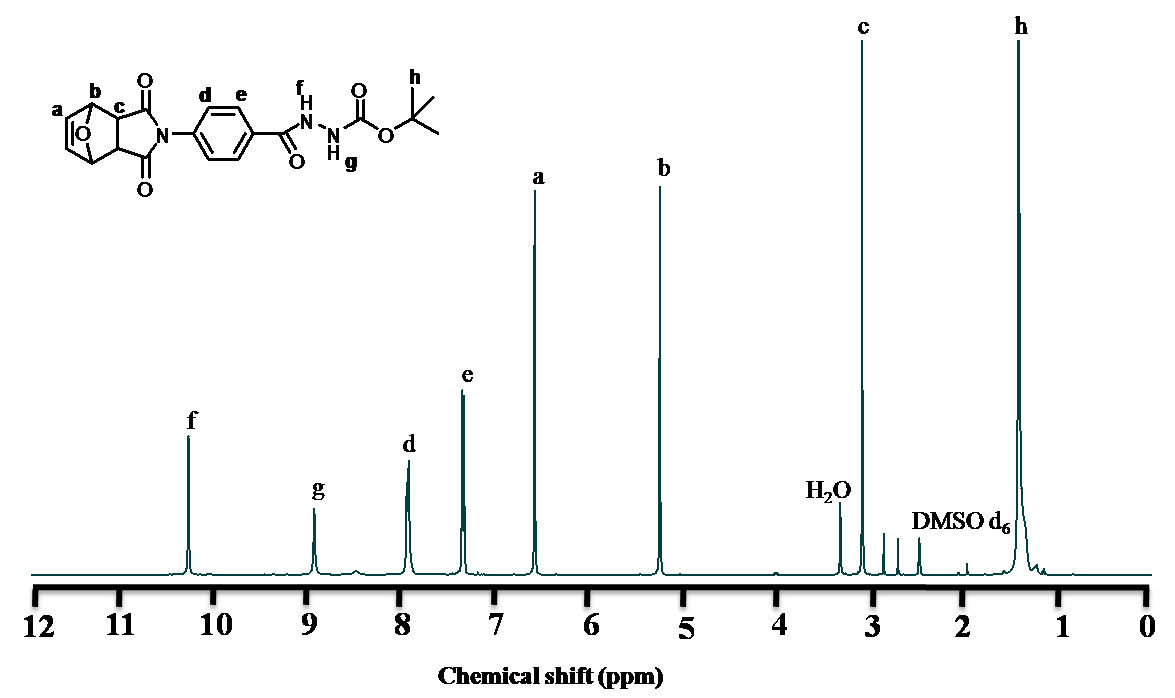
**

**Fig S18:** ^1^H NMR spectra for molecule **5** in DMSO-d6**.**

**
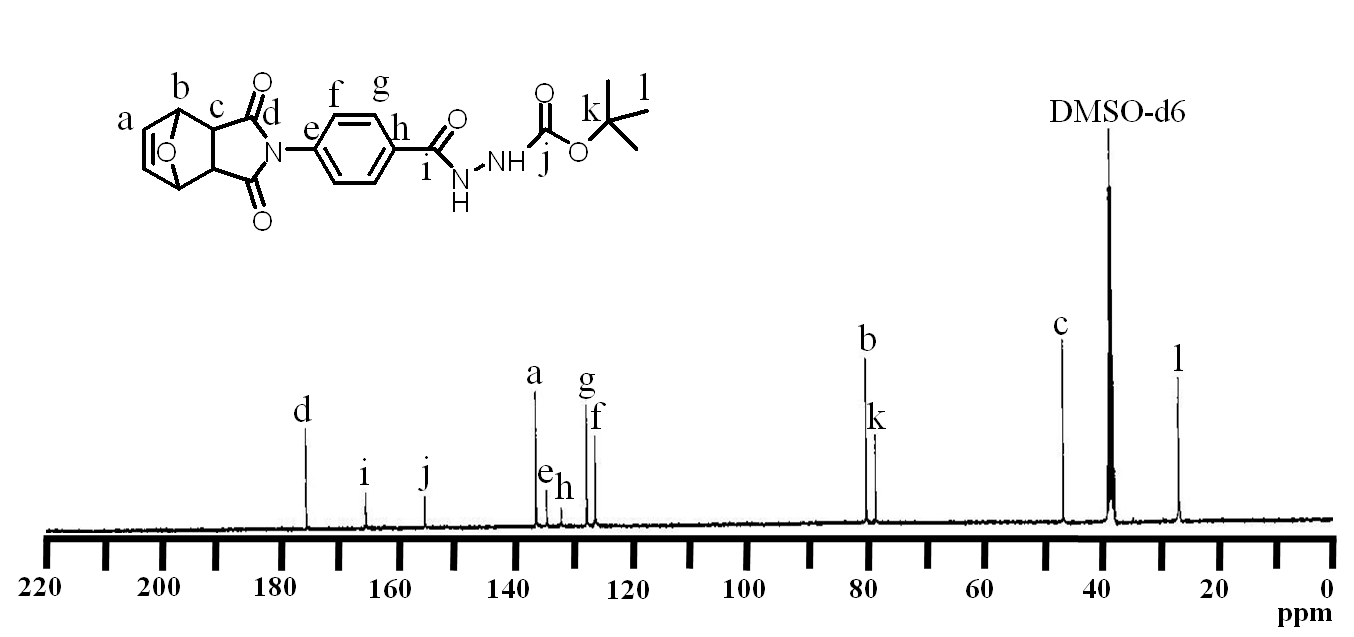
**

**Fig S19:** ^13^C NMR spectra for molecule **5** in DMSO-d6**.**

**
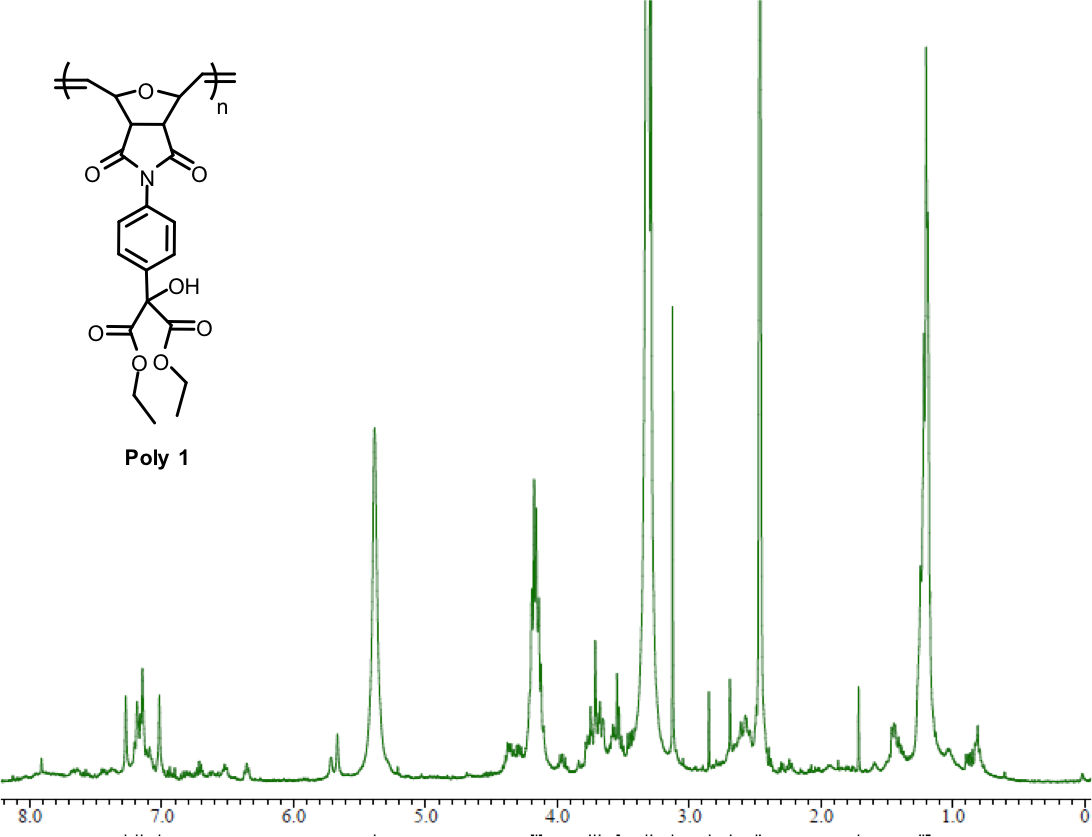
**

**Fig S20:** ^1^H NMR spectra for **Poly 1** in DMSO-d6**.**

**
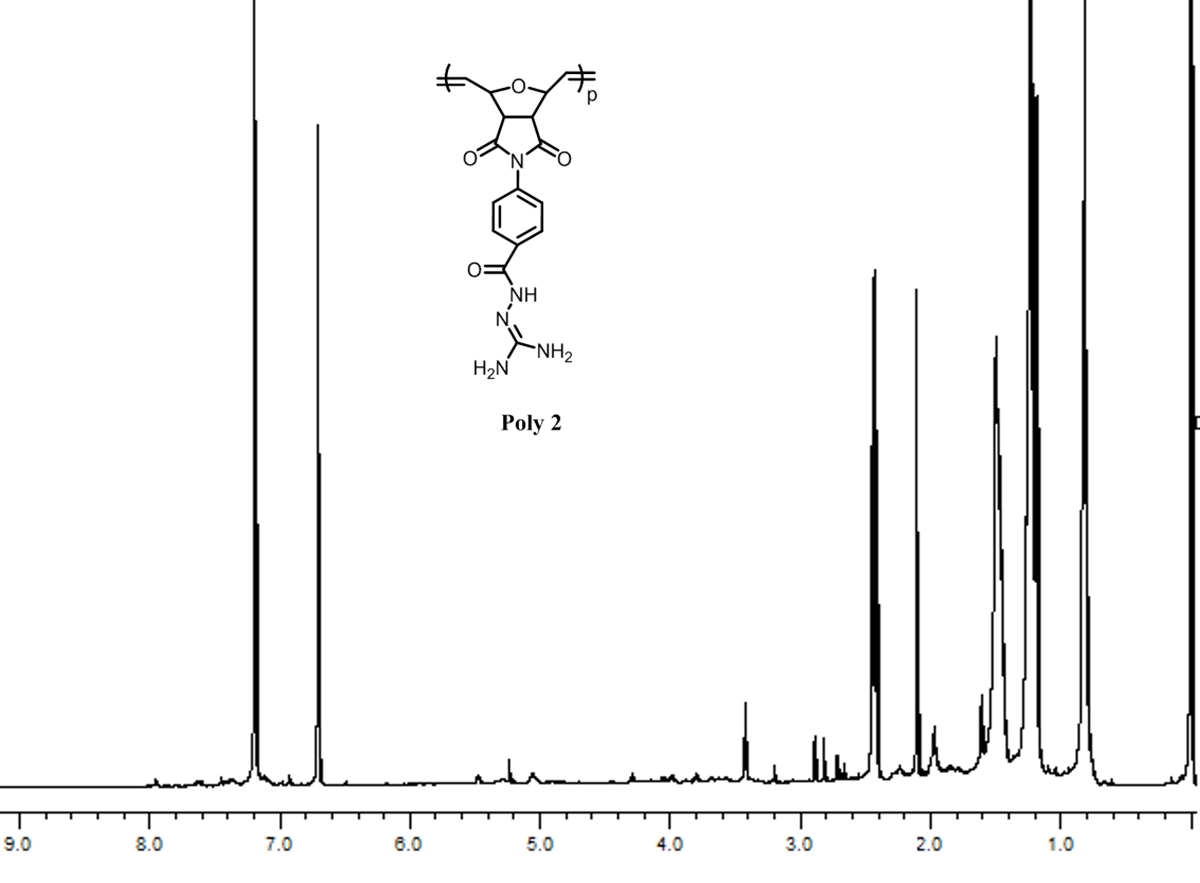
**

**Fig S21:** ^1^H NMR spectra for **Poly 2** in DMSO-d6**.**

**
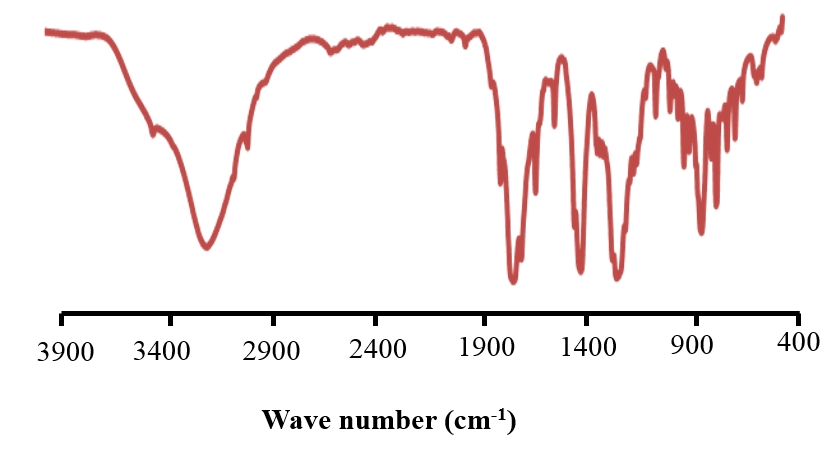
**

**Fig S22:** FT-IR spectra for molecule **4**.


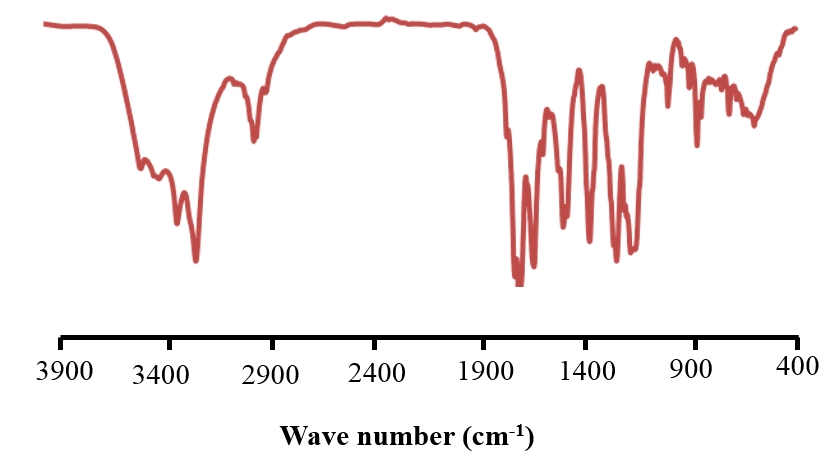


**Fig S23:** FT-IR spectra for molecule **5**.
